# Recent Advances in Experimental Burn Models

**DOI:** 10.3390/biology10060526

**Published:** 2021-06-12

**Authors:** Dandan Hao, Mahtab Nourbakhsh

**Affiliations:** Department of Geriatric Medicine, RWTH Aachen University Hospital, 52074 Aachen, Germany; dhao@ukaachen.de

**Keywords:** burn injury, thermal burns, chemical burns, radiation burns, tissue damage, burn wound progression, animal model, in vitro model

## Abstract

**Simple Summary:**

Human burns are diverse and the most difficult injuries to study in clinical settings. Numerous experimental burn models designed to study and compare different aspects of burns and their consequences and treatment are steadily progressing. This review summarizes the latest advances in experimental burn research as a guide to aid in the future design of studies.

**Abstract:**

Experimental burn models are essential tools for simulating human burn injuries and exploring the consequences of burns or new treatment strategies. Unlike clinical studies, experimental models allow a direct comparison of different aspects of burns under controlled conditions and thereby provide relevant information on the molecular mechanisms of tissue damage and wound healing, as well as potential therapeutic targets. While most comparative burn studies are performed in animal models, a few human or humanized models have been successfully employed to study local events at the injury site. However, the consensus between animal and human studies regarding the cellular and molecular nature of systemic inflammatory response syndrome (SIRS), scarring, and neovascularization is limited. The many interspecies differences prohibit the outcomes of animal model studies from being fully translated into the human system. Thus, the development of more targeted, individualized treatments for burn injuries remains a major challenge in this field. This review focuses on the latest progress in experimental burn models achieved since 2016, and summarizes the outcomes regarding potential methodological improvements, assessments of molecular responses to injury, and therapeutic advances.

## 1. Introduction

Various cross-species experimental burn models, including in vitro and animal models, have been established over the decades to study the etiology and mechanism of human burn injuries. Animal studies currently play a crucial role in biomedical research and remain the most frequently applied type of experimental model in burn research. However, the poor translation of data from animals into the human system has long been recognized. Furthermore, the application of large-scale damage, such as burns, to animals in research raises reasonable societal and ethical issues [1]. The general consensus is that the animal burden for scientific purposes must comply with the “3Rs”: replacement of animals by alternatives wherever possible, reduction in the number of animals used, and refinement of experimental conditions and procedures to minimize the harm to animals (https://arriveguidelines.org/arrive-guidelines, accessed on 11 June 2021). Concomitantly, humanized animal and pure human tissue burn models are increasingly emerging and promise to produce more clinically applicable data on the human inflammatory response to burn injury.

In general, the ultimate objectives of burn studies vary substantially, involving different aspects of tissue damage, inflammation, signal transduction, cell–cell interactions or new therapeutic products. Unlike clinical studies, experimental burn models were designed for comparative studies of burn consequences under defined or controlled conditions. However, the primary challenge of any experimental burn model remains the infliction of comparable and reproducible burn injuries in a set of parallel experiments. Therefore, continuous efforts are being made to advance existing models or establish new models, and to improve the reproducibility and validity of the results in different fields of burn research. This review outlines the major advances in the development and application of experimental burn models and the most relevant outcomes. The term “experimental” refers to controlled conditions of reproducible burn insults and thereby excludes clinical studies. Furthermore, this review focuses on the application of experimental burn models since 2016, as previous study models have been thoroughly summarized previously [2,3]. In particular, human cutaneous and corneal burn models have been improved to study local mechanisms of burn damage and progression. Humanized models have also been developed to study long-term systemic reactions to burns, molecular mechanisms of systemic inflammatory response syndrome (SIRS), and new therapeutic nanovehicles.

## 2. Burn Injuries

Since 2016, most experimental burn models have been established with thermal burns, including contact burns, scald burns, flame burns and inhalation burns (Table 1).

Chemical and radiation burns were studied in only 14 articles. Three main reasons for the superior prevalence of thermal burns in experimental burn models have been proposed. First, thermal burns are the most common type of human burn injuries that require admission to specialized burn centers. Approximately 86% of burn injuries are thermal burns; 43% are caused by fire or flame, 34% by hot fluids and 9% by hot objects. Only 4% of burns are caused by electricity, 3% by chemicals, and 7% by radiation or other sources. Accordingly, the consequences of chemical and radiation burns have been studied occasionally, but the main aim of these studies was to compare the consequences of these burns with those of thermal burns. Second, a variety of inexpensive and easy adjustable instruments are available for inflicting thermal burns at a controlled temperature, duration and location. Third, thermal burn devices are versatile and can be utilized on small and larger animals as well as on isolated tissue in vitro. Thermal contact burns are generally used to replicate small and local burns in studies of damage progression or the effects of topical drugs on burn wounds. However, scald burns are often used to inflict large area burns in animals and monitor the systemic response to burns. In addition, inhalation burns have been employed to inflict severe burns in larger animals and study systemic responses and multiple organ dysfunction.

### 2.1. Local Impacts

#### 2.1.1. Skin

In recent experimental settings, skin burn wounds have been inflicted by high temperatures, radiation alone, or both in combination. For thermal injuries, researchers usually determine a precise duration and temperature, which are adjusted to a particular animal model, the total surface area and the required degree of burn. Thus, the temperature and duration vary among reported studies, ranging from 54 to 330 °C for 4 s to 5 min depending on the animal model utilized. While thermal adjustments are easy to achieve, the infliction of radiation injuries is more complex. For instance, the dose and exposure time of gamma radiation and UV light vary significantly among experiments and individual settings. Therefore, most radiation-inflicted injury models require laborious pilot experiments to determine the unique adjustments needed for each experimental setting. Furthermore, human or porcine ex vivo and in vitro skin models have been established to study the immediate local response to thermal burns or novel treatments [44,49,50,51,52,73].

The progression of burn injuries is largely unpredictable, and many unknown factors significantly influence clinical outcomes. In current clinical practice, the course of treatments relies on a wait-and-see strategy to avoid unnecessary surgical procedures and the risk of infection, which may impair recovery. Therefore, a preliminary estimation of tissue damage progression is a decisive step in the treatment of burn patients. Indeed, burn wound progression is a well-documented phenomenon involving the successive progression of tissue damage into deeper layers of the primary exposed surface, even days after the burn injury itself. In animal models, tissue damage progresses for at least 3 days after burn injury, depending on the exposure temperature and time [46]. While the consequences of tissue damage may be diverse and complex, factors such as insufficient blood perfusion, hypercoagulation, necrosis, inflammatory responses, the formation of neutrophil extracellular traps (NETs) and changes in the wound microenvironment have been suggested to play additional roles [34,40]. On the other hand, the characteristics of the burn itself may affect the progression of tissue damage and thereby the healing of the burn wound. In an in vitro human cutaneous composite tissue model, flame burns caused an immediate deep burn, whereas contact burns tended to induce superficial damage that progressed deep into underlying tissue within 7 days after the burn [49,50].

Scalds are a subtype of contact burns caused by a hot liquid or steam. The majority of current scald burn models are based on the Walker–Mason burn model, which was initially developed to inflict large-area burn wounds in rats and expose them to bacterial infection. This model was successfully applied to monitor the systemic response or study novel local/systemic antimicrobial and antibiofilm therapies [21,22,23,24,25,26].

Another serious outcome of burn injuries is the formation of hypertrophic scars (HSs), which are characteristic of human but not animal skin. Many previous studies have attempted to establish hypertrophic scars in animal models. To date, three animal models have been reported to be moderately successful in the study of hypertrophic scar tissue. For instance, hypertrophic scarring was induced in rabbit ears by inflicting thermal contact burns [46,48]. In addition, in an immunodeficient mouse model, human hypertrophic scars were transplanted to study new treatments for scar tissue repair [5,30,31,32]. However, porcine models appear to represent the most relevant animals for mimicking human hypertrophic scars, as burn wounds in pigs tend to heal via the cumulative formation of fibrotic, hypercontracted and hyperpigmented tissue, a process similar to that leading to human hypertrophic scarring [38].

#### 2.1.2. Cornea

Corneal burns are very common in the workplace and are caused mainly by acidic and/or alkaline substances. Several animal models have been established to study corneal burn injuries. These models generally use a NaOH solution (1 N), which is directly applied to the center of the corneal surface in animals for 15–60 s [53,54,55,56,60,61,63]. Similar to the process in humans, the process of corneal alkali burn healing in animal models involves immediate local inflammation followed by fibrosis and neovascularization. Injury from a NaOH-induced corneal burn reaches the limbus and results in a depletion of limbal stem cells. Hence, animal corneal alkali burn models have also been used to study the pathogenesis of limbal stem cell deficiency (LSCD), which also results from chemical corneal injuries in humans. Rodent and rabbit models have frequently been used to understand the pathophysiology of corneal burn injuries and to develop novel therapeutic approaches. However, the corneas of rodents and rabbits are smaller and thinner than those of humans, and the risk of alkali-induced limbal damage is much higher in these small animals than in humans. Thus, the assessment of the healing response and the efficacy of investigative therapies may be inaccurate if rodent or rabbit models are used [56]. A novel dog model has been developed in which topical corneal alkali burns cause significant opacity and fibrosis without harming the limbus [63]. This model may facilitate the assessment of therapies for corneal fibrosis that do not involve LSCD.

### 2.2. Systemic Effects

Severe burn injuries not only affect the local wound site but can also elicit systemic reactions, cause dysfunction of multiple organs, and lead to significant morbidity and mortality. For experimental studies of systemic reactions, inhalation, scald, flame and radiation burn techniques are generally used to inflict severe burns in animal models that affect 15–40% of the total body surface area (TBSA). While burn conditions may differ among species, burns covering a minimum of 6% of the TBSA were sufficient to induce a systemic reaction in a mouse model [72].

The most commonly reported complication after severe burns is bacterial pneumonia. According to previous reports, severe burns cause pulmonary immunosuppression, which substantially increases the susceptibility of patients to opportunistic bacterial infections. Accordingly, several rodent models have been developed to study the mechanisms underlying pulmonary immunosuppression [70]. Experiments in a mouse model showed that burns of 20% TBSA led to significant hyperresponsiveness rather than immunosuppression in lung tissue [75]. Despite these similarities, animals and humans show significant differences in the course of recovery from severe burn injuries. Patients suffering from severe burns develop multiple organ dysfunction syndrome (MODS) and have extremely high rates of death within 2 weeks. However, MODS is highly difficult to establish in animals due to their remarkably fast recovery [79,81]. For instance, burn injuries affecting 60% of the TBSA result in a mortality rate of 30% to 40% in humans, but do not cause significant mortality in rodents. Indeed, protective mechanisms were suggested using a mouse model. For example, blood circulation is immediately differentially regulated in response to severe burns to protect important organs, such as the heart and brain [71]. The gastrointestinal tract subsequently becomes hypoxic and ischemic due to an insufficient blood supply. This process induces intestinal autophagy, which plays a protective role in intestinal injury after serious burn injury in mice [71]. Another study using a sheep burn model suggested that a three-stage coagulopathic response is induced after burn injuries of 40% TBSA, which result in either hyper- or hypocoagulation and a mortality rate of 30-40% in humans [67].

## 3. Responses to Burns

### 3.1. Local Response

Regardless of the species, the corporal response to burns and the healing of burns follow a course of hemostasis, inflammation, proliferation and remodeling. Ideally, this process ultimately leads to imperceptible scarring via minor fibrosis and insignificant contraction by fully preserving tissue architecture and function. However, undesirable wound tissue progression and excessive scar formation are very common in clinical practice and often involve later surgical treatments. Although the precise mechanisms of hypertrophic tissue expansion are not known, some implicated pathways have recently been extensively studied using experimental burn models (Table 2).

#### 3.1.1. Burn Wound Progression

Wound progression is characterized by a secondary wave of inflammation and necrosis adjacent to the primary wound site a few days after the insult. The previous hypothesis that wound progression results from surrounding bacterial contamination has long been disproven, and recent studies postulated a role for neutrophil activation and influx in controlling wound expansion. In this scenario, NETs are assembled through an active, generally suicidal process called NETosis, whereby neutrophils discharge net-like structures of DNA and histones as well as numerous different antimicrobial and proinflammatory granular proteins, including myeloperoxidase, elastase and cathepsin G. NETosis has been detected in burn wound tissue from both animal models and burn patients [34]. Neutrophil activation results in the production of proinflammatory cytokines, such as tumor necrosis factor-alpha (TNF-α), which may enhance ischemic tissue necrosis. TNF-α was shown to be upregulated 3 days after burn injury, and its neutralization has been shown to decrease burn wound progression in animal models [46]. In addition, IgM antibodies increase the burn wound depth, and the inhibition of IgM-mediated inflammation limits burn wound progression in a porcine model [40]. In a human composite tissue model, milder contact burns were more likely to inflict burn wound progression than severe flame burns [50]. Thus, thermal force gradients may also exert a relevant effect on burn wound expansion as a component of the inflammatory tissue environment.

#### 3.1.2. Hypertrophic Scarring

Hypertrophic scarring is a dermal fibroproliferative disorder characterized by excessive disposition of extracellular matrix (ECM). Numerous studies have reported the link between inflammation and fibrosis. Transforming growth factor beta (TGF-β) is the most potent cytokine for inducing fibrosis and scar formation. It is expressed and secreted by numerous cells, such as activated T cells, macrophages, neutrophils and platelets. In a porcine model, the TGF-β1 mRNA level was shown to be significantly increased in human HSs [38]. Other inflammatory cytokines, such as insulin-like growth factor (IGF-1), are also involved in ECM accumulation [38]. These observations led to the hypothesis that an exaggerated inflammatory phase promotes subsequent fibrosis and hypertrophic scarring. In addition, the proinflammatory chemokines interleuin (IL)-6 and IL-8 were shown to be substantially downregulated in mature human scars and keloids, confirming the importance of a postburn anti-inflammatory state [31].

Recent studies have also focused on proteins with possible modulatory effects on tissue fibrosis. Lumican is an ECM-secreted proteoglycan that has been reported to alleviate collagen fibrillogenesis in burn wounds [48]. The study used a rabbit ear model to investigate HS formation by primary fibroblasts isolated from patients, and the data indicated that HS tissue of burn patients exhibits a significantly lower level of lumican than unharmed cutaneous tissue [48]. In a mouse model of hypertrophic scarring using human skin samples, flightless I (Flii) was identified as a profibrotic protein that remodels actin filaments in HS and thereby contributes to excessive scar formation in both mice and humans [33].

#### 3.1.3. Corneal Neovascularization

The healthy cornea appears clear and expresses several antiangiogenic factors, including vascular endothelial growth factor-R1 (VEGF-R1), which contributes to the maintenance of a unique avascular status. A rabbit model of chemical corneal burns indicated the role of limbal stem cells in the initiation of neovascularization after burn injury [54]. In a mouse corneal burn model, corneal-limbal epithelial membrane protein-2 (EMP2) was identified as a promoter of pathological corneal neovascularization [60]. Studies using another mouse model showed that galectin-3 enhances corneal angiogenesis by modulating VEGF/VEGFR-2 signaling [59]. In addition, the crucial role of the Wnt/β-catenin signaling pathway in corneal angiogenesis was confirmed in a rat model of alkali-induced corneal burns [61].

### 3.2. Systemic Responses

Severe burns are defined as those that affect >20% TBSA and involve the skin and possibly other organs, such as the lungs or intestinal tract. This level of tissue destruction can result in a serious systemic response that affects nearly every other organ and is associated with a high risk of mortality without appropriate and timely intervention.

#### 3.2.1. Systemic Inflammatory Response Syndrome (SIRS)

SIRS is characterized by an abnormal increase in a subset of circulating cytokines in response to burns. However, the exact regulatory mechanisms that lead to cytokine release are not fully understood. Among these cytokines, brain natriuretic peptide (BNP) was observed to be expressed at high levels in patients with infectious or noninfectious SIRS, suggesting its central role [69]. Experimental rat and mouse models revealed that systemic BNP expression was selectively increased by burn injuries accompanied by SIRS compared with those not accompanied by SIRS [69]. Further studies indicated that BNP mediates SIRS by binding to the atrial natriuretic peptide receptor (NPRA) on macrophages and activating heat shock factor 1 (HSF-1).

#### 3.2.2. Pulmonary Immunosuppression

Pulmonary immunosuppression is an early response to severe burn injury that was recently studied in a mouse model. In this model, lipopolysaccharide (LPS) was applied intranasally in the presence or absence of scald burns. LPS induced neutrophil recruitment and NET formation, i.e., NETosis, in the airways. However, burn injury significantly reduced LPS-mediated leukocyte recruitment and NETosis in mice. This immunosuppression was accompanied by reduced levels of CCL2 and CCL3, and the suppression of LPS-mediated increases in IL-17A, IL-17C and IL-17E/IL17-25 levels in the airways [70].

#### 3.2.3. Systemic Myopathy

Skeletal muscle myopathy caused by proteolysis has been frequently reported as a complication of SIRS. Importantly, in this process, proteolytic pathways are persistently activated much later after burn injury, when the levels of inflammatory factors in plasma are significantly decreased. A mouse burn model revealed that the dysregulated expression of a membrane repair protein, mitsugumin 53 (MG53), plays a major role in burn-induced myopathy [50]. Furthermore, insulin increases MG53 expression, suggesting that burn-mediated MG53 downregulation may be caused by insulin insensitivity after burn injury [65].

#### 3.2.4. Intestinal Autophagy

Severe burns can lead to hypoxia/ischemia of the intestinal mucosa. Experimental studies using a mouse model of severe burns suggested that autophagy, a regulated process that mediates the degradation and recycling of cellular components, may be essential for cell survival after severe burns to limit tissue damage [71]. Successive studies of cellular signaling pathways in this mouse model have implicated the AMPK-mTOR signaling pathway in the activation of burn-induced intestinal autophagy [71].

## 4. Novel Therapeutic Approaches

The most common aim of experimental burn studies is to test and identify new potential therapeutic targets. Almost all burn-mediated alterations, such as coagulation, inflammation, angiogenesis, fibroplasia, contraction, remodeling and mechanical tension, are promising targets for new effective therapies. In addition, various techniques, such as surgical excision, laser therapy, cell therapy, nanocarrier delivery, irreversible electroporation and tissue engineering/scaffold implementation, have been advanced to support and enhance the healing of cutaneous (Table 3) and corneal burn wounds (Table 4). Animal models are still widely used, with rodents favored for mechanistic studies. However, human ex vivo models are gaining traction, with the advantage that they reduce and replace animal experiments and provide native skin tissue architecture to recapitulate important aspects of the human burn response [44,51,52,73].

### 4.1. Treatment of Cutaneous Burns

#### 4.1.1. Antinecrotic Strategies

Surgical debridement is an important step in the treatment of patients with deep dermal burns. The purpose is to remove necrotic and infectious material and to prepare tissue for skin grafting and definitive wound closure. A new hydrosurgical debridement system was developed for wound debridement in a rat burn model [10]. This hydrosurgical debridement system enabled less invasive and faster debridement, minimizing the risk of injury to healthy tissue and uncontrolled inflammation [10]. In addition, in a rat experimental burn model, bromelain was shown to reduce necrosis in the burn wound area and exhibited wound healing and tissue regeneration activities [9]. Another antinecrotic strategy was recently developed using bacterial nanocellulose-based wound dressing in a novel ex vivo model [52].

#### 4.1.2. Antimicrobial Strategies

Wound infection is the major cause of morbidity and mortality in burn patients. The effectiveness of traditional antibiotics is decreasing due to increasing bacterial resistance. Novel antibacterial antibodies, probiotics, antimicrobial peptides, chitosan, nanomaterials and silver-impregnated foam dressings have been evaluated as possible alternatives to traditional antibiotics in wound infection models [16,21,22,45,47,64,74]. Interestingly, a new therapeutic strategy based on bacterial interference was developed in an experimental rodent scald model [21]. *Lactobacillus plantarum*, a nonpathogenic microorganism with no virulence factors, was shown to play a protective role in methicillin-resistant *Staphylococcus aureus* superinfections [21]. 

#### 4.1.3. Anti-Inflammatory Strategies

Traditional anti-inflammatory agents, such as silver sulfadiazine, exhibit proven effectiveness in burn wound treatment, but they often cause adverse effects that limit their long-term use. Therefore, alternative natural compounds for the potential long-term treatment of burn damage and skin inflammation are in high demand. In experimental rodent models of contact or radiation burns, various nanoformulations based on silica, gold, polymers, and chitosan and lipid core nanocapsules have been investigated and shown to improve the activity of natural compounds by decreasing their instability or toxicity [9,17,18]. Indeed, hydrogels containing silibinin-loaded pomegranate oil-based nanocapsules exhibited more potent long-term anti-inflammatory effects on burn wounds than silver sulfadiazine [18].

#### 4.1.4. Cell Proliferation Strategies

Stem cell therapies are emerging as a promising strategy to treat burn wounds. Stem cells not only effectively accelerate burn wound healing but have also recently been utilized to generate skin appendages. Specifically, mesenchymal stem cells were shown to improve burn wound healing and the regeneration of sweat glands and skeletal muscle in rodent and porcine models [20]. In rodent models, adipose-derived stem cells were reported to increase burn wound healing and the regeneration of hair follicles or reduce scar formation [8,31]. Notably, a combination of cell therapy and nanotechnology using embryonic fibroblasts and graphene quantum dots was successfully applied in a rodent contact burn model [11]. Three additional compounds were tested for their proliferation-promoting effects on rodent burn models. Hesperidin was used to promote cell proliferation and neovascularization via the VEGF signaling pathway [19]. In addition, 4′,6,7-trimethoxyisoflavone (TMF) and glycitin were shown to improve burn wound healing by interacting with keratinocytes and fibroblasts via the TGF-β signaling pathway [7].

#### 4.1.5. Antiscarring Strategies

After scar tissue matures, it cannot be dispersed by healthy adjacent tissue. Therefore, effective strategies must be established to inhibit aberrant scar formation. However, most current animal studies on antiscarring treatments are based on transplanted matured human scar tissue for two practical reasons. First, rodent models do not develop HSs. Second, establishing models with pig and rabbit ears, which eventually develop HSs after 7–10 weeks, is highly time-consuming and expensive. Despite these limitations, pigs are ideal animals for studying physical scar therapies. Both ablative and nonablative laser therapies have been successfully utilized to improve the appearance and increase the pliability of scars for 35–84 days in pig scar models [35,37].

Studies using grafted human scars in rodent models revealed that an injection of adipose stem cells promotes the shrinkage of keloids/scars and improve the appearance of scars [30,31]. In addition, several combination drug therapies have been tested in similar experimental settings. For example, a combined intralesional injection of botulinum toxin type A (Botox A) and triamcinolone exerted beneficial effects on human scars grafted onto mice [32]. A combination of calcium channel blockers, steroids, and interferon reduced scarring in grafted human burn scar tissue in a mouse model [5]. In addition, novel electrospun microfibrous scaffolds and irreversible electroporation therapies have been shown to increase the elasticity of human burn scar tissue in rodents [4,14].

Pirfenidone is an FDA-approved antifibrotic drug for the treatment of idiopathic pulmonary fibrosis. A mouse model of deep partial thickness burns revealed the anti-inflammatory effect of pirfenidone [28,29]. Further experimental burn studies confirmed that the application of pirfenidone on cutaneous burn wound sites has an excellent scar-reducing effect [27].

### 4.2. Treatment of Corneal Burns

#### 4.2.1. Antiangiogenic Strategies

Anti-VEGF therapy is an FDA-approved and clinically successful treatment for corneal injury. However, adverse events, including systemic vascular toxicity, elevated intraocular pressure and cardiac complications, may occur. Clearly, the development of more rational anti-VEGF regimens and drugs that target other key factors and signaling pathways that mediate angiogenesis is important. Galectin-3 and EMP2 are novel modulators of VEGF signaling. As anti-VEGF agents, both were confirmed to inhibit corneal angiogenesis in murine corneal alkali burn models [59,60]. In a similar rat corneal burn model, angiogenic and inflammatory factors were significantly inhibited using humanized antibodies that block Wnt/β-catenin signaling [61]. Further investigations are warranted to determine the safety of these antiangiogenic agents in the treatment of corneal burn injuries.

#### 4.2.2. Antifibrotic Strategies

Suberanilohydroxamic acid is an approved drug for the therapy of patients with cutaneous T cell lymphoma. Its antifibrotic effect was previously reported in studies using human corneas in vitro and rabbits in vivo. However, in a subsequent study of corneal alkali burns in dogs, the beneficial effect of suberanilohydroxamic acid on corneal fibrosis was not confirmed [63]. Pirfenidone was also shown to enhance corneal wound healing not only by exerting antifibrotic effects but also by shortening the re-epithelialization time and exerting antiangiogenic effects [57,58,62].

#### 4.2.3. Re-Epithelialization

Tetrahedral framework nucleic acids (tFNAs) are new promising nanomaterials with potential positive effects on cell proliferation. Experiments in a rabbit corneal burn model confirmed that tFNAs improve corneal transparency and accelerate the re-epithelialization of injured corneal tissue [55]. In addition, a scaffold-based tissue engineering approach was developed for the regulation of key cellular events, such as proliferation and matrix synthesis, and the delivery of these engineered cell grafts to sites of tissue defects. Gelatin/ascorbic acid cryogels, as keratocyte carriers, were shown for the first time to exhibit improved abilities to enhance tissue matrix regeneration and maintain transparency, as well as to mitigate corneal damage [53].

## 5. Conclusions

Human burn injuries often impose severe functional, aesthetic and psychological burdens on patients; accordingly, these burdens have prompted numerous experimental burn studies aiming to develop new therapies. This review of burn research studies conducted within the past 5 years highlights two important limitations in this research field. First, the diversity of animal models and the poor translation of results into the human system may obscure the outcomes related to the assessment and treatment of human burn injuries. Second, inconsistencies in the technical equipment and experimental burn procedures used have resulted in numerous isolated reports with observations that cannot be compared.

To date, all animal burn studies and novel humanized animal studies have relied on standard pathological assessments at the final stage. In most instances, intermediate observations are lacking, which limits our understanding of burn wound progression and dynamics in different models. Considering that burn experiments on animals are cruel and less relevant to the human system, we were surprised to find more than 60 studies involving animal burn experiments that have been published since 2016. The true animal burden in these studies is difficult to estimate, as only a subset of relevant experiments are usually presented and the number of preliminary experiments remains unknown.

In general, ex vivo and in vitro models are useful alternatives to overcome this deficiency; however, the maximum life span of tissues in these models is two weeks. Future advances and improved protocols for the long-term maintenance of tissue cultures are likely to advance studies of burn wound dynamics in vitro. In the future, more state-of-the-art models are required to mimic the structure and function of human organs. Furthermore, advanced computer-modeling techniques (often referred to as in silico models) and studies with human volunteers may also follow.

Despite these limitations, an increasing number of reports are addressing novel therapeutic targets and strategies for burn wounds. These studies show the crucial roles of cytokines, chemokines and growth factors in the burn wound healing process and outcomes. In addition, stem cell therapy and new drug delivery and tissue engineering approaches have achieved significant advances, and provide a deeper understanding of the molecular mechanism underlying wound healing and an updated arsenal of technology for treatments. However, the clinical translation of this knowledge into effective treatments remains a challenge.

## Figures and Tables

**Table 1:** Experimental burn models utilized since 2016.

Topic	Species	Burn Injury	Human Relevance	References
Cutaneous injury	Rodents	Contact burn	Scar tissue	[4,5]
				[6,7,8,9,10,11,12,13,14,15,16]
		Radiation burn		[17,18,19]
		Scald		[20,21,22,23,24,25,26,27,28,29]
		Burn scar graft	Scar and keloid tissue	[30,31,32]
		Bleomycin-induced scar	Scar tissue	[33]
	Pigs	Contact burn	Eschar tissue	[34]
				[35,36,37,38,39,40,41,42,43,44]
	Rabbits	Contact burn		[45,46,47]
		Punch scar	Scar tissue	[48]
	Human	Flame burn	Composite cutaneous tissue	[49]
		Contact burn	Composite cutaneous tissue	[50,51,52]
Corneal injury	Rabbits	Chemical burn		[53,54,55,56,57,58]
	Rodents	Chemical burn		[59,60,61,62]
	Dogs	Chemical burn		[63]
Systemic injury involving lungs, intestines, muscles, etc.	Rodents	Radiation + scald burn		[64,65]
		Radiation + flame burn		[66]
		Inhalation + flame burns		[67]
		Scald burn	Humanized mice	[68]
				[69,70,71,72,73]
		Flame burn		[74]
		Contact burn		[75,76,77,78]
	Sheep	Inhalation + contact burns		[79]
	Pigs	Radiation burn		[80]
		Contact burn		[81]

**Table 2:** Signaling pathways evaluated in response to burns.

Class	Characteristic	Factors/Modulators	Pathways/Parameters
Cutaneous injury	Wound expansion	Neutrophils [34,46]	NETs and NETosis, TNF-α
		Natural IgM [40]	Ig-M-mediated pathway
		Thermal forces [50]	
	Hypertrophic scarring	Inflammatory cytokines [31,38]	TGF-β, IGF-1, IL-6, IL-8
		Lumican [48]	Integrinα_2_β_1_- FAK
		Flii [33]	Collagen I/III ratio, TGF-β_1_
Corneal injury	Neovascularization	Galectin-3 [59]	VEGF, bFGF
		EMP2 [60]	VEGF
		Epithelial cells [61]	Wnt/β-catenin, VEGF, TNF-α, ICAM-1
Severe injury	SIRS	Macrophages [69]	BNP/NPRA
	Pulmonary immunosuppression	Neutrophils [55]	NETs and NETosis
	Systemic myopathy	Myocyte membranes [65]	MG53
	Intestinal autophagy	Intestinal epithelium [71]	AMPK-mTOR

**Table 3:** Experimental therapeutic strategies for cutaneous burns.

Study Aim	Potential Therapeutic Targets	Route of Administration
Antinecrotic strategies	Hydrosurgical debridement [10]	Surgical excision
	Bacterial nanocellulose [52]	Dressing
Antimicrobial strategies	Silver-impregnated foam dressings [22]	Dressing
	Chitosan/PVA membranes [45]	Dressing
	Antimicrobial peptide: DGL13K [16]	Vehicle
	Anti-pcrV IgY antibodies [74]	Subcutaneous injection
	*Lactobacillus plantarum* [21]	Subcutaneous injection
	ZnO_2_ + nanoparticles [47]	Topical
Anti-inflammatory strategies	Silibinin-loaded pomegranate oil + nanocapsules [17]	Topical
	Fumarate + poly nanofibers [18]	Topical
	Bromelain + chitosan nanofibers [9]	Topical
	IgM-N2 peptide [40]	Intravenous infusion
Strategies for promoting cell proliferation	Embryonic fibroblasts + graphene quantum dots [11]	Subcutaneous injection
	BM-MSCs [80]	Intramuscular injection
	Human BM-MSCs [20]	Subcutaneous injection
	ASCs [8]	Intradermal injection
	Hesperidin [19]	Oral
	TNF + glycitin [7]	Unknown
Antiscarring strategies	Irreversible electroporation [14]	Electroporation
	Pulsed dye laser, CO_2_ laser [35,37]	Focused light
	CCBs, steroids and interferon [5]	Intralesional injection
	Steroids and Botox A [32]	Intralesional injection
	Chyle fat [30]	Intralesional injection
	Human BM-MSCs [31]	Intraperitoneal injection
	Electrospun microfibrous scaffolds [4]	Surgical insertion
	Ad-lumican vectors [48]	Intralesional injection
	FnAb: neutralizing antibody against Flii [33]	Subcutaneous injection

**Table 4:** Experimental strategies for the treatment of corneal burns.

Study Aim	Potential Therapeutic Targets	Route of Administration
Antiangiogenic strategies	Galectin-3 inhibitor [59]	Ophthalmic (eye drops)
	EMP2 antibody blockade [60]	Subconjunctival injection
	Humanized antibody H1L1 [61]	Subconjunctival injection
Antifibrotic strategies	Suberanilohydroxamic acid [63]	Topical
	Pirfenidone [57,58,62]	Topical/eye drops/contact lens
Re-epithelialization	Gelatin/ascorbic acid cryogels [53]	Surgical insertion
	tFNAs [55]	Topical

## Data Availability

Not applicable.
